# Antimicrobial Activity of Aztreonam-Avibactam and Other β-Lactamase Inhibitor Combinations Tested Against Enterobacterales Isolates from Pediatric Patients from United States Medical Centers (2019–2023)

**DOI:** 10.3390/antibiotics14111107

**Published:** 2025-11-03

**Authors:** Helio S. Sader, Marisa L. Winkler, Krisztina M. Papp-Wallace, Rodrigo E. Mendes, Mariana Castanheira

**Affiliations:** Element Iowa City (JMI Laboratories), 345 Beaver Kreek Centre, Suite A, North Liberty, IA 52317, USA

**Keywords:** aztreonam-avibactam, ceftazidime-avibactam, meropenem-vaborbactam, imipenem-relebactam, cefiderocol, children, carbapenem-resistant Enterobacterales

## Abstract

**Objective:** To evaluate the antimicrobial susceptibility of Enterobacterales isolated from pediatric patients. **Methods:** A total of 5723 isolates were consecutively collected (1/patient) from pediatric patients (<18 years old [yo]) from 82 United States medical centers in 2019–2023 and susceptibility-tested by broth microdilution method. Susceptibility was stratified by infection type and patient age: ≤1 yo (*n* = 2275), 2–5 yo (*n* = 1130), 6–12 yo (*n* = 1213), and 13–17 yo (*n* = 1105) and compared to adults (18–64 yo; *n* = 17,712). **Results:** Pediatric isolates were mainly from pneumonia (21.8%), bloodstream (BSI; 15.3%), and urinary tract infection (UTI; 51.8%). Aztreonam–avibactam, ceftazidime–avibactam, and meropenem–vaborbactam were active against ≥99.4% of ceftriaxone-nonsusceptible (99.4–100.0% susceptible), multidrug-resistant (MDR; 99.7–100.0% susceptible), and ESBL producer (99.7–100.0% susceptible) isolates from pediatric patients. Susceptibility to imipenem–relebactam varied from 97.1% (ceftriaxone-nonsusceptible) to 100.0% (ESBL producers). Ceftolozane–tazobactam showed good activity against ESBL producers (91.8% susceptible), but limited activity against ceftriaxone-nonsusceptible (75.8% susceptible) and MDR isolates (80.9% susceptible). The MDR phenotype varied from 14.3% (13–17 yo) to 19.7% (6–12 yo) among pediatric isolates (15.8% overall) and was 20.7% among adult Enterobacterales. Carbapenem resistance rates were markedly lower in pediatric (0.1%) isolates compared to adult isolates (1.3%). The ESBL profiles were similar among pediatric and adult isolates; 90.1% of ESBL producers from pediatric patients and 88.5% from adults carried a CTX-M +/− an OXA-1/30 gene. **Conclusions:** Antimicrobial resistance was generally lower among Enterobacterales from pediatric patients compared to adults. ESBL-producing Enterobacterales, mainly CTX-M, remain an important cause of infection in children. Aztreonam–avibactam, ceftazidime–avibactam, and meropenem–vaborbactam were highly active against isolates from both pediatric and adult population.

## 1. Introduction

Antibiotic resistance in Enterobacterales species has been increasing at an alarming pace for many years and of particular concern is the emergence and rapid dissemination of Enterobacterales resistant to third-generation cephalosporins and carbapenems [1].

Enterobacterales represent a major cause of healthcare-associated infections (HAIs) and community-acquired infections in pediatric patients. Antimicrobial resistance among Enterobacterales species began increasing rapidly in the 1990s, mainly due to the extensive spread of extended-spectrum β-lactamases (ESBLs), which encompass a large number of enzymes that hydrolyze third-generation cephalosporins and other broad-spectrum β-lactam agents [2,3,4]. ESBL-encoding genes are most frequently carried on mobile genetic elements (such as plasmids or transposons), and can disseminate very rapidly and cause resistance to all β-lactams except carbapenems, cephamycins, and the newer β-lactamases inhibitor combinations (BLICs) [5]. Resistance to broad-spectrum cephalosporins and piperacillin–tazobactam can also be caused by genes encoding AmpC cephalosporinases (AmpC), which can be located in the chromosome or in a plasmid. Notably, ESBLs and AmpC may confer carbapenem resistance when associated with alteration or loss of porins [6]. Moreover, since the 2000s, a fast global dissemination of carbapenem-resistant Enterobacterales (CRE) has been facilitated by mobile genetic elements carrying carbapenemase genes, such as *Klebsiella pneumoniae* carbapenemase (KPC), metallo-β-lactamases (MBLs), and oxacillinase 48 (OXA-48)-like enzymes [7]. CREs often carry other plasmid-encoded genes targeting different classes of antimicrobial agents, making them multidrug-resistant (MDR) [5].

The prevalence of CRE infections increased markedly in the United States (US) in the late 1990s and early 2000s, and these infections are associated with increased mortality and morbidity [8,9]. The National Healthcare Safety Network (NHSN) of the US Centers for Disease Control and Prevention (CDC) reported that the frequency of CRE among Enterobacterales increased from 1.2% in 2001 to 4.2% in 2011 [7]. NHSN data have also shown that the organisms implicated in HAIs and their antimicrobial resistance profiles vary greatly between adult and pediatric patients [10]. Thus, despite increased attention to ESBL producers and CRE in the last two decades, limited data is available on the frequency and epidemiology of infections caused by these organisms in US pediatric population.

Antimicrobial treatment options for infections caused by CRE and MDR Enterobacterales are still limited [11]. Although recently approved BLICs and cefiderocol represented significant progress in the treatment of CRE infections, resistance to these agents is emerging among CREs in the US and very limited data are available for the pediatric population [12,13]. Aztreonam–avibactam was approved by the European Medicines Agency (EMA) in the European Union in April 2024 for treatment of adults with complicated intra-abdominal infection (cIAI), complicated urinary tract infection (cUTI), hospital-acquired pneumonia, including ventilator-associated pneumonia, and infections due to aerobic Gram-negative bacteria in adults with limited treatment options (https://www.ema.europa.eu/en/news/new-antibiotic-fight-infections-caused-multidrug-resistant-bacteria; accessed on 25 August 2025) and more recently (February 2025) by the US FDA for treatment of adults with complicated IAI. This agent combines the stability against hydrolysis by metallo-β-lactamases (MBLs) provided by aztreonam with the protection against serine β-lactamases, including extended-spectrum β-lactamases (ESBL), chromosomal derepressed AmpC, and KPCs, provided by avibactam [13,14]. In the present study, we evaluated the activities of aztreonam–avibactam, ceftazidime–avibactam, meropenem–vaborbactam, imipenem–relebactam, ceftolozane–tazobactam, and comparators against Enterobacterales isolates-caused infection in pediatric patients from US medical centers from 2019 to 2023.

## 2. Results

**Figure 1:**
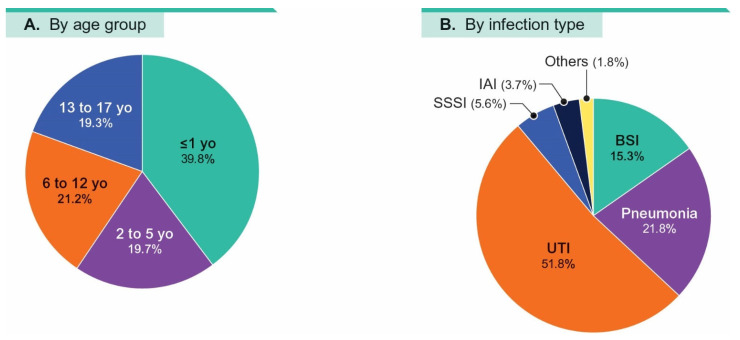
Distributions of patients by age group (**A**) and infection type (**B**). Abbreviations: yo, years old; BSI, bloodstream infection; UTI, urinary tract infection; SSSI, skin-and-skin structure infection; IAI, intra-abdominal infection.

Almost 40% (39.8%) of pediatric isolates were from patients ≤1 yo, and each of the other three age groups (2–5 yo, 6–12 yo, and 13–17 yo) had approximately 20% of isolates (19.3% to 21.2%; Figure 1A). Moreover, pediatric isolates were mainly from bloodstream (BSI; 15.3%), pneumonia (21.8%), and UTI (51.8%; Figure 1B).

**Figure 2:**
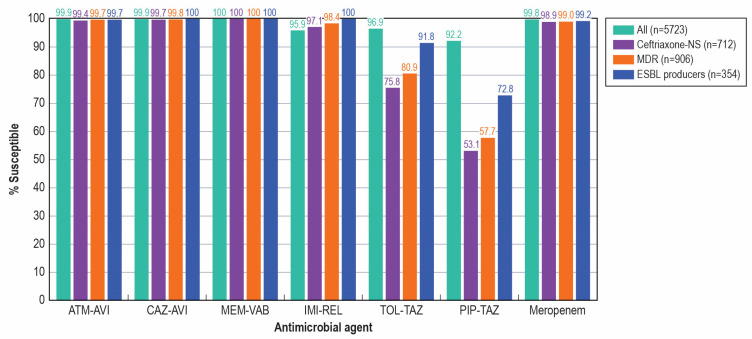
Antimicrobial activity of β-lactamase inhibitor combinations against Enterobacterales and resistant subsets from pediatric patients. Abbreviations: ATM–AVI, aztreonam–avibactam; CAZ–AVI, ceftazidime–avibactam; MEM–VAB, meropenem–vaborbactam; IMI–REL, imipenem–relebactam; TOL–TAZ, ceftolozane–tazobactam; PIP–TAZ, piperacillin–tazobactam. NS, nonsusceptible; MDR, multidrug-resistant.

Aztreonam–avibactam, ceftazidime–avibactam, and meropenem–vaborbactam were highly active against isolates from both pediatric and adult population. Aztreonam–avibactam inhibited 99.9% of pediatric and adult isolates, including 100.0% of isolates from the 2–5 yo and 6–12 yo age groups, at ≤4 mg/L, which is the susceptible breakpoint established by the US FDA (https://www.fda.gov/drugs/development-resources/aztreonam-and-avibactam-injection, accessed 25 August 2025), CLSI [15], and EUCAST [16] (Table 1). Ceftazidime–avibactam was active against >99.9% of pediatric isolates and 99.7% of adult isolates and meropenem–avibactam was active against 100.0% of pediatric isolates and 99.6% of adult isolates (Table 1 and Figure 2). Imipenem–relebactam and ceftolozane–tazobactam were slightly less active, with susceptibility rates of 95.9% and 96.9% for pediatric isolates and 93.1% and 93.9% for adult isolates, respectively (Table 1 and Figure 2). Piperacillin–tazobactam was the least active BLIC tested, with susceptibility rates of 92.2% for pediatric and 88.0% for adult isolates (Table 1 and Figure 2). Meropenem, ceftriaxone, and gentamicin were active against 99.8%, 87.6%, and 92.1% of pediatric isolates, respectively (Table 1).

Aztreonam–avibactam, ceftazidime–avibactam, meropenem–vaborbactam, and meropenem were active against >99% of pediatric isolates independent of infection source. Moreover, susceptibility rates for comparator agents were similar among infection types (Table 2).

The activities of the BLICs against resistant subsets from pediatric patients are shown in Table 3 and Figure 2. Aztreonam–avibactam, ceftazidime–avibactam, and meropenem–vaborbactam exhibited ≥99.4% activity against ceftriaxone-nonsusceptible (99.4–100.0% susceptible), MDR (99.7–100.0% susceptible), and ESBL producers (99.7–100.0% susceptible). Susceptibility to imipenem–relebactam varied from 97.1% (ceftriaxone-nonsusceptible) to 100.0% (ESBL producers). Ceftolozane–tazobactam showed good activity against ESBL producers (91.8% susceptible), but limited activity against ceftriaxone-nonsusceptible (75.8% susceptible) and MDR isolates (80.9% susceptible). Piperacillin–tazobactam displayed limited activity against all three resistant subsets (53.1–72.8% susceptible; Table 3 and Figure 2).

**Figure 3:**
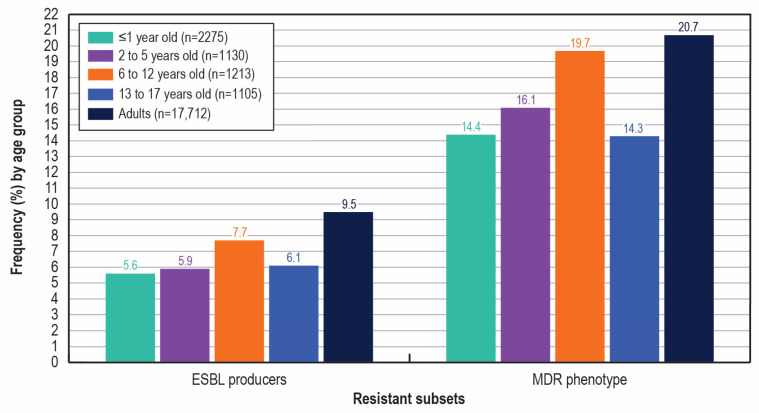
Frequency of extended-spectrum β-lactamase (ESBL) producers and isolates with a multidrug-resistant (MDR) phenotype stratified by age group. Abbreviations: ESBL, extended-spectrum β-lactamase; MDR, multidrug-resistant.

Susceptibility rates were comparable among pediatric age groups and generally higher for pediatric isolates compared to adult isolates (Table 1). Notably, the frequency of ESBL producers and MDR phenotypes were slightly higher among the 6–12 yo age group when compared to the other pediatric age groups, and higher among adult isolates compared to isolates from pediatric patients. The frequencies of ESBL producers varied from 5.6% (≤1 yo) to 7.7% (6–12 yo) among pediatric age groups (6.2% overall) and was 9.5% among adult isolates. The occurrences of MDR phenotypes varied from 14.3% (13–17 yo) to 19.7% (6–12 yo) among pediatric isolates (15.8% overall) and was 20.7% among adult isolates (Figure 3). Moreover, the carbapenem resistance rate was markedly lower among pediatric isolates (0.1%) compared to adult isolates (1.3%).

**Figure 4:**
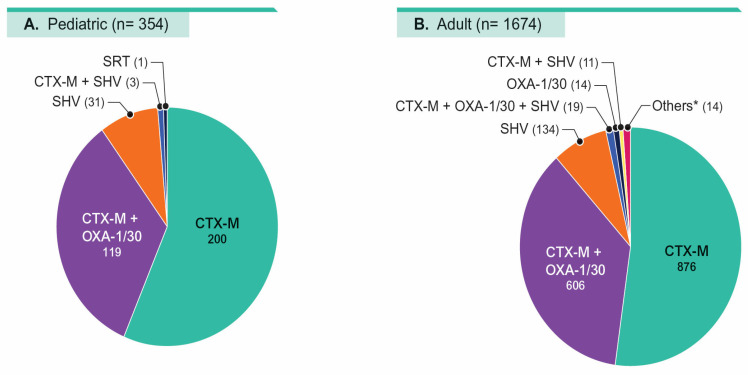
Distribution of extended-spectrum β-lactamase (ESBL) types among pediatric (**A**) and adult (**B**) population. * Includes TEM (6), SRT (3), CTX-M + OXA-1/30 + TEM (2), PER (2), and CTX-M + TEM (1).

The ESBL profiles were similar among pediatric and adult isolates. Briefly, 90.1% of ESBL-producing isolates from pediatric patients and 88.5% from adults carried a CTX-M +/− an OXA-1/30 gene, and most of the remaining isolates in each group carried an SHV gene (Figure 4).

## 3. Discussion

Infections caused by MDR Enterobacterales represent a major concern and increasing problem in children [18,19]. MDR infections are related to increased morbidity and mortality, time of hospitalization, and costs when compared to infections caused by susceptible organisms, and β-lactamase production represents the main cause of increasing antimicrobial resistance in Enterobacterales [3,20].

In the present study, the antimicrobial susceptibility of 5723 Enterobacterales isolates collected from pediatric patients hospitalized in US medical centers was evaluated and compared to 17,712 isolates from adults collected from the same hospitals during the same time period. Overall, 15.8% of isolates from pediatric patients exhibited an MDR phenotype and 6.2% produced at least one ESBL. The newer BLICs, aztreonam–avibactam, ceftazidime–avibactam, and meropenem–vaborbactam, as well as meropenem alone, were highly active against pediatric isolates (≥99.8% susceptibility), and these compounds retained potent activity against ceftriaxone-nonsusceptible (98.9–100.0% S), MDR (99.0–100.0% S), and ESBL producer (99.2–100.0% S) isolates.

We were not able to compare our results with those from other investigators since limited data exists on resistance profiles for bacterial pathogens isolated from children in US medical centers, especially regarding the susceptibility of MDR isolates and the activity of the BLICs released for clinical use in the last decade [13]. NHSN reported the frequency and antimicrobial susceptibility of organisms recovered from selected HAI among pediatric patients from 2545 US medical centers in 2015–2017 [10]. Enterobacterales accounted for >30% of the organisms overall, including around 60% of organisms recovered from catheter-associated UTI, 30% of organisms from ventilator-associated pneumonia and surgical infections, and 25% of organisms from central line-associated infections. The report; however, provided limited data on antimicrobial susceptibility of the organisms [10].

Logan et al. [4] evaluated the prevalence of CRE from pediatric patients by using the antimicrobial susceptibilities of Enterobacterales reported by 300 laboratories that participated in The Surveillance Network (TSN; Eurofin-Medinet, Herndon, VA, USA) between January 1999 and July 2012. Among the results from the 316,253 isolates analyzed, only 266 (0.08%) isolates were identified as CRE. The study found that the CRE infection rate increases were highest for *Enterobacter* species, blood culture isolates, and isolates from intensive care units, increasing from 0.0% in 1999–2000 to 5.2%, 4.5%, and 3.2%, respectively, in 2011–2012. In contrast, results from our investigation showed that the CRE infection rate remains low among a large collection of contemporary isolates from 82 US medical centers. The main limitation of the investigation performed by Logan et al. is that the investigators relied on the antimicrobial susceptibility results provided by the participant centers, which were mainly generated by automated systems [4].

In another investigation, Logan et al. [21] evaluated 225 isolates with an ESBL or CRE phenotype recovered from pediatric patients hospitalized between January 2011 and April 2015 at three hospitals in the Chicago area. Overall, 90.7% of isolates carried a *bla* gene, and the most common was *bla*_CTX-M-1_ group (49.8%). Only 1.8% of isolates carried a carbapenemase gene: three *bla*_KPC_ and one *bla*_IMP_ [21]. Our results corroborate the results of this investigation by showing the predominance of *bla*_CTX-M_ among ESBL producers.

In a previous study, we evaluated the antimicrobial susceptibility of 4724 Enterobacterales collected in 2011–2015 from pediatric patients hospitalized in 82 US medical centers through the same surveillance program utilized in the current investigation [22]. Susceptibility results of the pediatric population for ceftazidime–avibactam (>99.9%) and meropenem (99.6%) were similar to those obtained in the present study (>99.9% and 99.8%, respectively), and susceptibility to piperacillin–tazobactam (94.1%), ceftriaxone (88.6%), and levofloxacin (93.4%) were slightly higher in 2011–2015 compared to 2019–2023 (92.4%, 87.6%, and 90.8%, respectively) [22].

One important finding of this investigation was the fact that antimicrobial resistance was generally lower among Enterobacterales isolated from pediatric patients compared to adults hospitalized in the same hospitals at the same time. The frequency of key resistance phenotypes, such as MDR, ESBL producers, and CRE, were markedly lower among pediatric patients compared to adults. Because limited data is available on antimicrobial resistance in children, data generated from the adult population may be used to guide empiric antimicrobial therapy. However, the differences in antimicrobial susceptibility between Enterobacterales from these two populations, as observed in this investigation, emphasize the importance of conducting antimicrobial resistance surveillance focused specifically on pediatric patients.

In conclusion, our results showed that aztreonam–avibactam, ceftazidime–avibactam, and meropenem–vaborbactam are highly active against Enterobacterales caused-infection in pediatric patients from US medical centers. ESBL-producing Enterobacterales remain an important cause of infection in children and CTX-M is by far the most common ESBL in this population. Additionally, the rate of CRE infections among children appears to be lower than among adults, and meropenem remained very active against Enterobacterales caused-infection in children in the US medical centers evaluated in this investigation.

## 4. Material and Methods

### 4.1. Organism Collection

The bacterial isolates were consecutively collected in 2019–2023 via the International Network for Optimal Resistance Monitoring (INFORM) Surveillance Program [12]. Each participating center was requested to provide a certain number of consecutive patient unique isolates from designated infection types collected during a specific period of the year, independent of the patient age. Demographic information, such as culture date, hospitalization unit, and patient age and sex, were evaluated on isolates exhibiting uncommon susceptibility profile to check for possible outbreaks. A total of 43,325 Enterobacterales isolates were collected from 82 US medical centers during the investigation period. Among those, 5723 (13.2%) were from pediatric patients (<18 yo) and 17,712 were from adult patients (18 to 64 yo); patient age was not provided for 1322 patients. The medical centers were distributed in 38 states from all 9 US Census Divisions. Only isolates determined to be significant by local criteria as the reported probable cause of infection were included in the investigation. Antimicrobial susceptibility results from isolates from pediatric patients were evaluated and compared to those from adults. For the pediatric population, susceptibility results were stratified by patient age as follows: ≤1 yo (2275 isolates), 2–5 yo (1130), 6–12 yo (1213), and 13–17 yo (1105). Where necessary, standard biochemical tests and the MALDI Biotyper (Bruker Daltonics, Billerica, MA, USA) confirmed species identification.

### 4.2. Susceptibility Testing

Isolates were susceptibility-tested at a monitoring laboratory (Element Iowa City [JMI Laboratories]) by the reference broth microdilution method as described by the Clinical Laboratory Standards Institute (CLSI) [15]. Aztreonam–avibactam, ceftazidime–avibactam, imipenem–relebactam, ceftolozane–tazobactam, and piperacillin–tazobactam were tested with a β-lactamase inhibitor at a fixed concentration of 4 mg/L; meropenem–vaborbactam was tested with vaborbactam at a fixed concentration of 8 mg/L [15,23]. MIC panels were inoculated using Sensititre AIM™ Automated Inoculation Delivery System (ThermoFisher Scientific; Lenexa, KS, USA). MIC values were interpreted according to CLSI and/or US FDA breakpoint criteria unless otherwise noted. Isolates were categorized as MDR according to criteria defined in 2012 by the joint European and US Centers for Disease Control [23]. These criteria define MDR as nonsusceptible to ≥1 agent in ≥3 antimicrobial classes and extensively drug resistant (XDR) as susceptible to ≤2 classes. The ESBL phenotype was defined for *Escherichia coli*, *Klebsiella oxytoca*, *K. pneumoniae*, and *Proteus mirabilis* as an MIC value ≥2 mg/L for ceftriaxone, ceftazidime, and/or aztreonam [17]. Categorical interpretations followed CLSI [17] and/or US FDA criteria FDA (https://www.fda.gov/drugs/development-resources/aztreonam-and-avibactam-injection, accessed 21 August 2025), unless otherwise noted. Results were stratified by age group, infection type, and resistant subsets.

### 4.3. Screening for β-Lactamases

Enterobacterales isolates displaying an ESBL phenotype were screened for β-lactamase-encoding genes using next-generation sequencing. *Citrobacter* spp. and *Enterobacter* spp. were screened only when resistant to cefepime. Total genomic DNA was prepared using the KingFisher Cell and Tissue DNA kit (ThermoFisher Scientific, Waltham, MA, USA) or the MagMax DNA Multi-Sample Ultra 2.0 extraction kit (ThermoFisher) on a KingFisher Flex Magnetic Particle Processor (ThermoFisher). DNA libraries were constructed using either the Nextera XT library construction protocol and index kit or the Illumina DNA prep (Illumina, Inc., San Diego, CA, USA). Sequencing was performed on either a NextSeq 1000 Sequencer (Illumina) using NextSeq 1000/2000 P2 Reagents (300 cycles) or a MiSeq Sequencer with a MiSeq Reagent Kit v3 (600 cycles) (Illumina). The generated FASTQ files were assembled using SPAdes Assembler and subjected to proprietary software (Element Iowa City [JMI Laboratories]) for screening of β-lactamase genes [24]. Libraries were normalized using the bead-based normalization procedure (Illumina) and sequenced on MiSeq. FASTQ files were assembled using de novo assembler SPAdes 3.9.0 and subjected to a proprietary software (Element Iowa City [JMI Laboratories]) for screening of β-lactamase genes [25].

## Figures and Tables

**Table 1:** Antimicrobial activity of aztreonam–avibactam and comparator agents tested against Enterobacterales isolates stratified by patient age group.

	% Susceptible by Patient Age Group ^a^ (No. of Isolates in Parenthesis):					
Organism/Antimicrobial Agent	≤1 yo	2–5 yo	6–12 yo	13–17 yo	All Peds	Adults ^b^
Enterobacterales	(2275)	(1130)	(1213)	(1105)	(5723)	(17,712)
Aztreonam–avibactam ^c^	99.9	100.0	100.0	99.9	99.9	99.9
Ceftazidime–avibactam	100.0	99.9	100.0	99.9	>99.9	99.7
Meropenem–vaborbactam	100.0	100.0	100.0	100.0	100.0	99.6
Imipenem–relebactam	98.1	92.7	95.3	94.6	95.9	93.1
Ceftolozane–tazobactam	96.5	97.7	96.5	97.6	96.9	93.9
Piperacillin–tazobactam	92.0	93.5	90.3	93.4	92.2	88.0
Ceftriaxone	87.6	89.2	84.5	89.2	87.6	81.5
Cefepime	94.0	93.9	91.5	93.0	93.3	88.5
Meropenem	99.8	99.7	100.0	99.8	99.8	98.6
Levofloxacin	94.2	88.8	87.5	89.4	90.8	82.1
Gentamicin	93.8	90.8	90.4	91.8	92.1	91.2
Amikacin	96.0	95.0	94.6	96.0	95.5	95.0

^a^ Criteria as published by CLSI [17]. ^b^ 18–64 years old. ^c^ Susceptible at ≤4 mg/L https://www.fda.gov/drugs/development-resources/aztreonam-and-avibactam-injection (accessed on 21 August 2025). Abbreviations: yo, year(s) old; Peds, pediatric.

**Table 2:** Antimicrobial activity of aztreonam–avibactam and comparator agents tested against Enterobacterales isolates from pediatric patients stratified by infection type.

	% Susceptible by Infection Type ^a^ (No. of Isolates in Parenthesis):					
Organism/Antimicrobial Agent	BSI	IAI	Pneumoniae	SSSI	UTI	Others
Enterobacterales	(874)	(214)	(1245)	(321)	(2967)	(102)
Aztreonam–avibactam ^b^	100.0	100.0	99.8	100.0	>99.9	99.9
Ceftazidime–avibactam	99.9	100.0	100.0	99.7	100.0	100.0
Meropenem–vaborbactam	100.0	100.0	100.0	100.0	100.0	100.0
Imipenem–relebactam	98.6	99.1	98.1	92.4	93.6	100.0
Ceftolozane–tazobactam	96.6	93.5	96.5	94.1	97.8	96.1
Piperacillin–tazobactam	92.3	88.3	90.2	89.0	93.6	95.1
Ceftriaxone	87.3	86.4	86.0	87.2	88.4	88.2
Cefepime	92.8	93.5	93.6	96.9	92.8	96.1
Meropenem	99.5	99.5	99.8	99.7	>99.9	100.0
Levofloxacin	90.1	92.1	92.0	93.8	89.8	99.0
Gentamicin	93.7	91.1	92.3	96.0	91.0	97.1
Amikacin	95.7	94.9	96.2	96.6	95.0	99.0

^a^ Criteria as published by CLSI [17]. ^b^ Susceptible at ≤4 mg/L (https://www.fda.gov/drugs/development-resources/aztreonam-and-avibactam-injection, (accessed on 21 August 2025). Abbreviations: BSI, bloodstream infection; IAI, intra-abdominal infection; SSSI, skin-and-skin structure infection, UTI, urinary tract infection.

**Table 3:** Antimicrobial susceptibility of multidrug-resistant (MDR) and extended-spectrum β-lactamase (ESBL) producers from pediatric patients.

Resistant Subset (No.)/Antimicrobial Agent	MIC (mg/L)		Susceptibility per CLSI and/or US FDA)		
	50%	90%	% Susceptible	% Intermediate	% Resistant
Ceftriaxone-NS (712)					
Aztreonam–avibactam	0.06	0.5	99.4	0.3	0.3
Ceftazidime–avibactam	0.25	0.5	99.7		0.3
Meropenem–vaborbactam	0.03	0.06	100.0	0.0	0.0
Imipenem–relebactam	0.12	0.5	97.1 ^b^	2.6	0.3
Ceftolozane–tazobactam	1	16	75.8	7.3	16.9
Piperacillin–tazobactam	8	128	53.1	12.8	34.1
Ceftriaxone	>8	>8	0.0	6.6	93.4
Cefepime	4	>32	45.8	12.5	41.7
Meropenem	0.03	0.12	98.9	0.6	0.6
Levofloxacin	0.25	16	64.6	5.9	29.5
Gentamicin	0.5	>16	70.1	0.7	29.2
Amikacin	2	8	86.5	7.4	6.0
MDR (906) ^a^					
Aztreonam–avibactam	0.06	0.5	99.7	0.1	0.2
Ceftazidime–avibactam	0.12	0.5	99.8		0.2
Meropenem–vaborbactam	0.03	0.03	100.0	0.0	0.0
Imipenem–relebactam	0.12	0.25	98.4 ^b^	1.3	0.2
Ceftolozane–tazobactam	0.5	8	80.9	5.8	13.2
Piperacillin–tazobactam	8	128	57.7	12.5	29.8
Ceftriaxone	>8	>8	34.0	2.4	63.6
Cefepime	1	>32	61.6	8.6	29.8
Meropenem	0.03	0.06	99.0	0.4	0.6
Levofloxacin	0.5	16	61.0	8.1	30.9
Gentamicin	1	>16	57.4	0.9	41.7
Amikacin	2	8	85.3	9.2	5.5
ESBL producers (354) ^c^					
Aztreonam–avibactam	0.06	0.12	99.7	0.0	0.3
Ceftazidime–avibactam	0.12	0.5	100.0		0.0
Meropenem–vaborbactam	0.03	0.03	100.0	0.0	0.0
Imipenem–relebactam	0.12	0.25	100.0 ^b^	0.0	0.0
Ceftolozane–tazobactam	0.5	2	91.8	2.3	5.9
Piperacillin–tazobactam	4	32	72.8	14.2	13.0
Ceftriaxone	>8	>8	0.0	0.3	99.7
Cefepime	>32	>32	10.2	13.8	76.0
Meropenem	0.03	0.06	99.2	0.6	0.3
Levofloxacin	1	16	41.8	8.2	50.0
Gentamicin	1	>16	53.1	0.6	46.3
Amikacin	4	8	78.8	12.4	8.8

^a^ Organisms include *Citrobacter amalonaticus* (1), *C. amalonaticus/farmeri* (1), *C. freundii* species complex (47), *C. koseri* (3), *E. cloacae* species complex (135), *Escherichia coli* (459), *Hafnia alvei* (1), *Klebsiella aerogenes* (34), *K. oxytoca* (47), *K. pneumoniae* (110), *Morganella morganii* (1), *Pluralibacter gergoviae* (1), *Proteus mirabilis* (4), *Raoultella ornithinolytica* (1), *Serratia marcescens* (59), and unspeciated *Raoultella* (2). ^b^ All Enterobacterales species were included in the analysis, but CLSI excludes *Morganella*, *Proteus*, and *Providencia* species. ^c^ Organisms include *Citrobacter freundii* species complex (3), *C. koseri* (2), *Enterobacter cloacae* species complex (26), *Escherichia coli* (237), *Klebsiella oxytoca* (5), *K. pneumoniae* (79), *Raoultella ornithinolytica* (1), and *Serratia marcescens* (1). Abbreviations: CLSI, Clinical Laboratory Standards Institute; NS, nonsusceptible; MDR, multidrug-resistant; ESBL, extended-spectrum β-lactamase.

## Data Availability

The original contributions presented in this study are included in the article. Further inquiries can be directed to the corresponding author.
